# Sirtuin-3 (SIRT3), a Novel Potential Therapeutic Target for Oral Cancer

**DOI:** 10.1002/cncr.25676

**Published:** 2010-11-29

**Authors:** Turki Y Alhazzazi, Pachiyappan Kamarajan, Nam Joo, Jing-Yi Huang, Eric Verdin, Nisha J D'Silva, Yvonne L Kapila

**Affiliations:** 1 >Department of Periodontics and Oral Medicine, School of Dentistry, University of MichiganAnn Arbor, Michigan; 2 Gladstone Institute of Virology and Immunology, University of CaliforniaSan Francisco, California; 3 Department of Pathology, University of MichiganAnn Arbor, Michigan

**Keywords:** SIRT3, sirtuins, carcinogenesis, oral cancer, squamous cell carcinoma

## Abstract

**BACKGROUND:**

Several sirtuin family members (SIRT1-7), which are evolutionarily conserved NAD-dependent deacetylases, play an important role in carcinogenesis. However, their role in oral cancer has not yet been investigated. Therefore, the objective of this study was to investigate whether sirtuins play a role in oral cancer carcinogenesis.

**METHODS:**

The expression levels of all sirtuins in several oral squamous cell carcinoma (OSCC) cell lines were compared with normal human oral keratinocytes and observed that SIRT3 was highly expressed. Therefore, tissue microarrays were used to evaluate the clinical relevance of this overexpression. SIRT3 down-regulation in OSCC cell proliferation and survival was investigated and analyzed by using cell-proliferation and cell-viability assays. Ionizing radiation and cisplatin were used to investigate whether SIRT3 down-regulation could increase the sensitivity of OSCC to both treatments. To further assess the in vivo role of SIRT3 in OSCC carcinogenesis, a floor-of-mouth oral cancer murine model was used to study the effect of SIRT3 down-regulation on OSCC tumor growth in immunodeficient mice.

**RESULTS:**

The current results demonstrated for the first time that SIRT3 is overexpressed in OSCC in vitro and in vivo compared with other sirtuins. Down-regulation of SIRT3 inhibited OSCC cell growth and proliferation and increased OSCC cell sensitivity to radiation and cisplatin treatments in vitro. SIRT3 down-regulation also reduced tumor burden in vivo.

**CONCLUSIONS:**

The current investigation revealed a novel role for SIRT3 in oral cancer carcinogenesis as a promoter of cell proliferation and survival, thus implicating SIRT3 as a new potential therapeutic target to treat oral cancer. **Cancer 2011**. © 2010 American Cancer Society.

**Oral** cancer is the eighth most common cancer worldwide, and oral squamous cell carcinoma (OSCC) accounts for >90% of all oral malignancies.^1^ The 5-year survival rates for patients with OSCC range from 34% to 62.9%, and those rates have not changed for decades.^2^ This underscores the need for new therapeutic targets to treat oral cancer. Recently, it was reported that several members of the sirtuin family (SIRT1-7), the human homologues of the Sir2 gene in yeast, play an important role in carcinogenesis.^3^ Sirtuins function either as nicotinamide adenine dinucleotide (NAD)-dependent deacetylases or as ADP-ribosyl transferases, which explains their involvement in a diversity of cellular functions, including regulation of oxidative stress, increasing genomic stability, cell survival, development, metabolism, aging, and longevity.^4^, ^5^ SIRT1, the best characterized family member, targets several key regulators that affect carcinogenesis, such as the tumor suppressor p53, the DNA repair protein Ku70, and the proapoptotic protein forkhead box O (FOXO) protein.^3^, ^6^ Current literature supports a prosurvival role for SIRT1 in colon, breast, and lung cancers through 1 or more of these previously mentioned targets.^3^ In contrast, other reports indicate that SIRT1 may act as a tumor suppressor.^7^, ^8^

Although much is known about SIRT1, less is known about other mammalian Sir2 homologues, such as SIRT3. SIRT3, which is the only member linked to longevity in humans,^9^-^11^ is a mitochondrial protein^12^-^18^ that is overexpressed in and associated with lymph node-positive breast cancer.^19^ Moreover, during stress, SIRT3 levels increase, protecting cells from apoptosis. Thus, like SIRT1, SIRT3 may bind and deacetylate Ku70, promoting Ku70-Bax interactions and attenuating apoptosis in cardiomyocytes.^20^ In addition, mitochondrial SIRT3 is required for Nampt, a stress and diet-responsive regulator of mitochondrial NAD^+^ levels, to promote cell survival during genotoxicity.^21^ More recently, it was reported that SIRT3 abrogates p53 activity, thus preventing growth arrest and senescence in bladder carcinoma cells.^22^ These findings suggest a role for SIRT3 in carcinogenesis; however, to our knowledge, the role of sirtuins has not been investigated in oral cancer. Therefore, the objective of the current study was to elucidate the role of sirtuins in oral cancer.

## MATERIALS AND METHODS

### Cell Lines and Culture

The human OSCC cell line HSC-3 was provided by Randy Kramer (University of California-San Francisco, San Francisco, Calif), and the OSCC cell lines UM-SCC-1 and UM-SCC-17B were provided by Tom Carey (University of Michigan, Ann Arbor, Mich). Primary human keratinocytes were derived from normal gingival tissues that were discarded from periodontal surgical procedures and were approved by the University of Michigan Institutional Review Board. OSCC cells were maintained in Dulbecco modified Eagle medium (DMEM) (Gibco, Carlsbad, Calif) supplemented with 10% fetal bovine serum and 1% penicillin/streptomycin. Primary human oral keratinocytes were maintained in EpiLife medium (Cascade Biologics, Portland, Ore).

#### Transient transfection

Cells were transiently transfected with small interfering RNA (siRNA) (150 nM; a pool of 3 target-specific siRNA) against SIRT3 or with a nontargeting control (Santa Cruz Biotechnology, Santa Cruz, Calif) in serum-free medium that contained Lipofectamine Plus (Invitrogen, Carlsbad, Calif). Transfection efficiency was assessed by Western blot analysis.

#### Stable transfection

UM-SCC-17B cells were transduced with SIRT3-short hairpin RNA (shRNA) (sc61555-vs) or scrambled-shRNA (sc-108084; both from Santa Cruz Biotechnology) lentiviral particles in 0.5 mL of serum-free media, then selected in 10 μg/mL puromycin for an additional 10 days (sc-108071; Santa Cruz Biotechnology). Surviving cell colonies were picked and propagated before testing for SIRT3 expression.

#### Cell-proliferation and colony-formation assays

To determine the effect of sirtinol, nicotinamide (NAM), ionizing radiation (IR), and cisplatin on cell proliferation, the QUANT Cell Proliferation Assay Kit was used according to manufacturer's instructions (Invitrogen). For colony-formation assays, OSCC cells were transfected as described above and were cultured for 1 week. Colonies were stained with 0.5% crystal violet, and the colonies that contained >50 cells were counted.

#### Apoptosis cell death detection by enzyme-linked immunosorbent assay

To measure apoptosis in vitro, a DNA-fragmentation enzyme-linked immunosorbent assay (ELISA) was used according to the manufacturer's instructions (Roche Diagnostics, Indianapolis, Ind).

#### Immunoblot analysis

To evaluate the expression levels of sirtuins in OSCC cells compared with normal primary human keratinocytes or the transfection/transduction efficiency of cells, cells were treated as described above and in figure legends, washed once with phosphate-buffered saline, and lysed in RIPA buffer (R0278, Sigma) that contained 1% protease inhibitor cocktail (P8340, Sigma) on ice for 30 minutes. Lysates were adjusted for protein concentration with the BCA protein assay kit (Bio-Rad, Hercules, Calif). Lysate proteins were resolved by SDS-PAGE and transferred to Immobilon-P membranes (Millipore, Billerica, Mass). Western blot analysis was performed with various primary antibodies and horseradish peroxidase-conjugated antirabbit or antimouse IgG antibodies, and blots were developed with the ECL-Plus detection system (Pierce, Rockford, Ill). Antibodies for SIRT1 (sc-15404) and SIRT2 (sc-20966) were obtained from Santa Cruz Biotechnology. The SIRT3 antibody (2627) was obtained from Cell Signaling Technology (Beverly, Mass). Antibodies to SIRT4 (IMG-3580), SIRT5 (IMG-479), and SIRT7 (IMG-425) were obtained from Imgenex (Bhubaneshwar, India). The SIRT6 antibody (AP6245a) was obtained from ABGENT (San Diego, Calif). To demonstrate equal protein loading, membranes were stripped and reprobed with an anti-β-actin antibody (sc-1615; Santa Cruz Biotechnology).

#### Tissue microarrays

Immunohistochemical analyses were performed to determine the expression of SIRT3 and SIRT7 in human normal and OSCC tissues using OSCC tissue microarrays (OR601 and HN241; US Biomax, Inc., Rockville, Md) and the Histostatin Kit (95-6143; Zymed Laboratories, South San Francisco, Calif) according to the manufacturer's instructions. Antibodies to SIRT3 (AP6242a) and SIRT7 (AP6246a) were obtained from ABGENT. Staining intensities were graded in a blinded manner as either low or high by a pathologist.

#### Immunodeficient mouse model of human head and neck squamous cell carcinoma

Three-week-old athymic nude mice (NCr-nu/nu strain; National Cancer Institute, Frederick, Md) that weighed between 20 and 25 g were anesthetized by intraperitoneal injection with 100 mg/kg ketamine and 10 mg/kg xylazine. The human OSCC cell line UM-SCC-17B that stably expressed SIRT3-shRNA or scrambled-shRNA was grown to 70% confluence before injection. We used a murine floor-of-mouth model, which we previously optimized to produce 4-mm to 5-mm tumors, corresponding to a palpable tumor volume of 35 mm^3^ to 60 mm^3^, within approximately 2 to 4 weeks after the injection.^23^ In brief, cells were suspended in DMEM, chilled on ice, and resuspended in an equal volume of growth factor-reduced Matrigel (BD Biosciences, San Jose, Calif; catalog no. 354230) to a final concentration of 2.5 × 10^5^/0.1 mL. The cell/Matrigel solution was chilled on ice before injection, and each animal was injected with an equal number of OSCC cells (2.5 × 10^5^) submucosally in the floor of the mouth. After 3 weeks, the mice were euthanized, and a digital caliper was used to determine the tumor volume using the formula *a* × *a* × *b*/2, where *a* is the smaller dimension.

### Statistical Analysis

Values were expressed as means ± standard deviation. Intergroup differences were determined by using a 2-way ANOVA and the Scheffe multiple-comparison test. Statistical significance was defined as *P ≤ .05, **P ≤ .01, and ***P ≤ .001. For tissue microarray analyses, the chi-square test was used. For the in vivo studies, independent *t* tests with unequal variances were used. All experiments were repeated at least 3 times.

## RESULTS

### SIRT3 Is Overexpressed in Oral Squamous Cell Carcinomas

**Figure 1:**
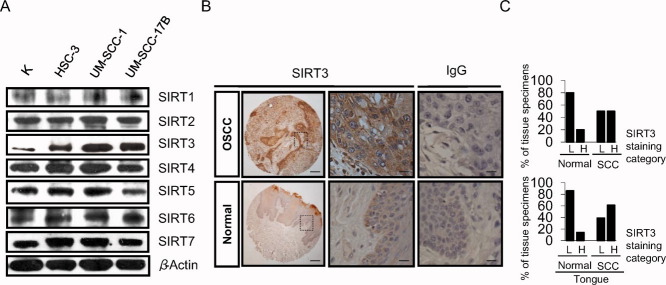
Sirtuin-3 (SIRT3) is overexpressed in oral squamous cell carcinoma (OSCC). (A) Immunoblots reveal the levels of sirtuins (SIRT1-7) in the OSCC cell lines HSC-3, UM-SCC-1, and UM-SCC-17B and in normal human oral keratinocytes (K). β-Actin served as loading control. (B) These representative samples show (*Top*) SIRT3 expression levels in OSCC (tongue) and (*Bottom*) in normal tissues. Immunoglobulin G (IgG) served as a negative control. Scale bars = 200 μm for low-magnification photomicrographs (left column); 50 μm for high-magnification photomicrographs (middle and right columns). (C) These charts illustrate the percentage of normal and OSCC tissue specimens that expressed SIRT3 (*Top*) in all samples and (*Bottom*) in tongue samples, as determined by immunohistochemical staining. Note that SIRT3 staining intensities were designated as low (L) or high (H) from the data provided in Table 1.

To determine whether sirtuins play a role in OSCC, we examined the protein levels of all sirtuins (SIRT1-7) in several OSCC cell lines (HSC-3, UM-SCC-1, and UM-SCC-17B) and compared those cells with normal primary human oral keratinocytes (Fig. 1A). Only SIRT3 and, to a lesser extent, SIRT7 were overexpressed in all 3 cell lines compared with primary keratinocytes. To further examine the in vivo and clinical relevance of SIRT3 and SIRT7, immunohistochemical analyses were performed for both sirtuins using tissue microarrays of OSCCs. In all, 52 samples were analyzed, including 42 malignant tumor samples and 10 normal tissue samples. Grade 1, 2, and 3 tumors from the tongue, cheek, gingiva, lip, and oral mucosa were analyzed along with normal tissues from the tongue, palate, and gingiva (Table 1). Staining intensity was assessed as either low or high (Table 1). SIRT3 expression was significantly higher in OSCC tissues compared with normal tissues (*P* ≤ .05) (Fig. 1B, Table 1), whereas SIRT7 expression levels did not differ significantly (data not shown). SIRT3 staining intensity data from Table 1 are illustrated in Figure 1C. SIRT3 exhibited an opposite pattern of expression between OSCC and normal tissues (Fig. 1C, top). Furthermore, because the tongue accounts for 30% of oral malignancies,^1^ we specifically examined tongue samples separately. SIRT3 staining intensity was significantly higher in OSCC tongue samples compared with normal tongue tissue samples (*P* ≤ .04) (Fig. 1C, bottom; Table 1).

**Table 1:** Correlation of Sirtuin-3 Expression and Clinicopathologic Variables in Normal and Oral Squamous Cell Carcinoma Tissues

	Total No. of Tissue Specimens	SIRT3 Staining Intensity: No. of Tissue Specimens (%)		*P*^a^
		Low	High	
Normal	10	8 (80)	2 (20)	
Tumor	42	21 (50)	21 (50)	.05 (Normal vs tumor)^b^
**Normal**	10			
**Tongue**	7	6 (86)	1 (14)	
**Tumor**	42			
**Tongue**	26	10 (39)	16 (61)	.04 (Normal tongue vs tumor tongue)^b^

SIRT3 indicates sirtuin-3.

a Statistical analysis: chi-square test.

b Significant difference (*P* ≤ .05).

### The Sirtuin Inhibitors Sirtinol and Nicotinamide Inhibit Cell Growth and Proliferation and Induce Apoptosis

**Figure 2:**
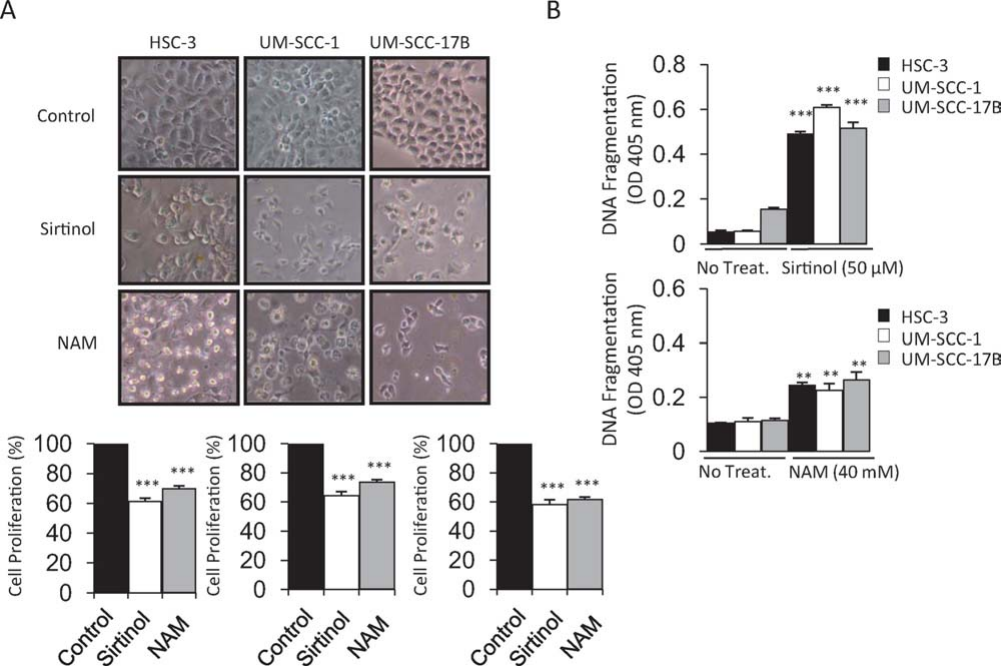
The sirtuin inhibitors sirtinol and nicotinamide (NAM) inhibit cell growth and proliferation and induce apoptosis. (A) Shown are (*Top*) phase-contrast images and (*Bottom*) histograms that reveal the morphology of cell growth and cell proliferation (%) of the HSC-3, UM-SCC-1, and UM-SCC-17B oral squamous cell carcinoma (OSCC) cell lines after treatment with sirtuin inhibitors sirtinol (50 μM) or NAM (40 mM) for 16 hours and 24 hours, respectively (original magnification, ×100). (B) Cell death-detection ELISA assays were used to measure DNA fragmentation in cells after treatment with (*Top*) sirtinol and (*Bottom*) NAM as indicated. **P ≤ .01; ***P ≤ .001; OD, optical density.

After we established that sirtuins (and specifically SIRT3) were associated with OSCC, we explored the role of sirtuins in modulating OSCC cell growth and proliferation. To this end, we tested the commonly used sirtuin inhibitors sirtinol and nicotinamide (NAM), which inhibit cell growth in breast and lung cancers.^24^, ^25^ Both inhibitors inhibited cell growth and proliferation in OSCC cells (Fig. 2A). In addition, both inhibitors induced apoptosis in OSCC cells compared with untreated controls, as determined by cell-death ELISA assays, which are used to measure DNA fragmentation (Fig. 2B).

### SIRT3 Down-Regulation Inhibits Cell Growth and Proliferation and Promotes Apoptosis in Oral Squamous Cell Carcinoma Cells

**Figure 3:**
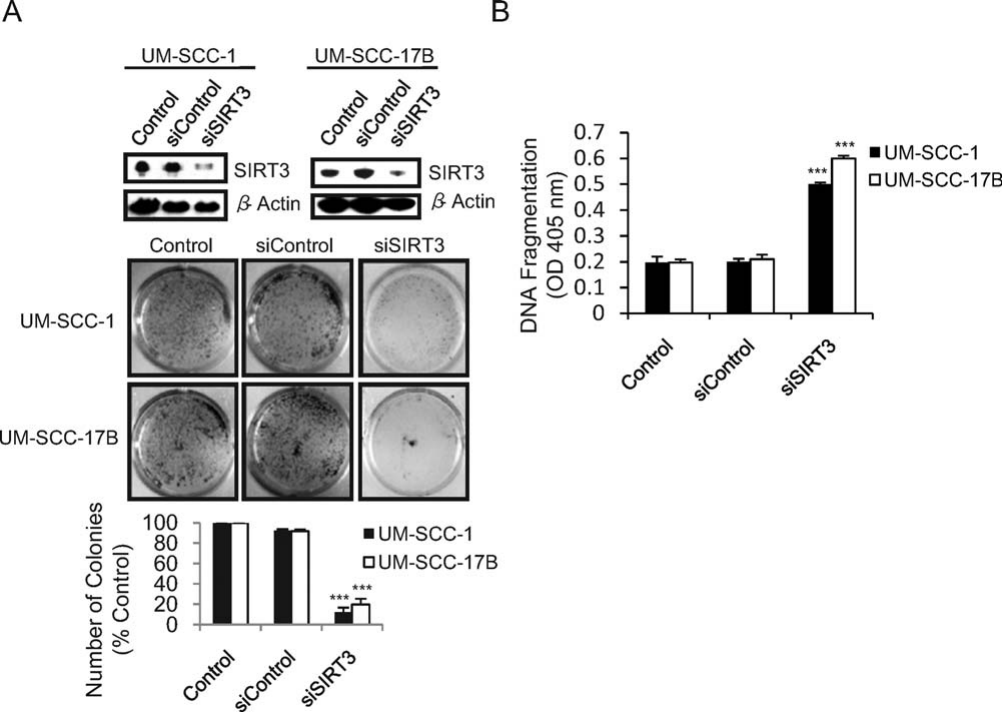
Sirtuin-3 (SIRT3) down-regulation inhibits cell growth and proliferation and promotes apoptosis in oral squamous cell carcinoma (OSCC) cells. (A) (*Top*) Immunoblots reveal the transfection efficiency of SIRT3 in the UM-SCC-1 and UM-SCC-17B OSCC cell lines 36 hours after transfection with SIRT3 small interfering RNA (siSIRT3) or nontargeting control (siControl) (150 nM). β-Actin served as loading control. (*Middle*) Cells were transfected as indicated and were cultured for 1 week, then stained with crystal violet, and photographed. (*Bottom*) Numbers of colonies are presented as the percentage of colonies obtained relative to controls. (B) Cell death-detection ELISA assays were used to measure DNA fragmentation in the cells after transfection. **P ≤ .01; ***P ≤ .001; OD, optical density.

To gain further insight into the effects of SIRT3 on OSCC cell survival and proliferation, we performed colony-formation assays in the context of SIRT3 inhibition with siRNA. Like in the cell growth studies, SIRT3 down-regulation inhibited colony formation in OSCC cells compared with controls (Fig. 3A). Furthermore, down-regulation of SIRT3 also promoted apoptosis in OSCC cells, mimicking the effect of sirtinol and NAM on these cells (Fig. 3B).

### SIRT3 Down-Regulation Enhances the Sensitivity of Oral Squamous Cell Carcinoma Cells to Radiation-Induced and Cisplatin-Induced Cytotoxicity

**Figure 4:**
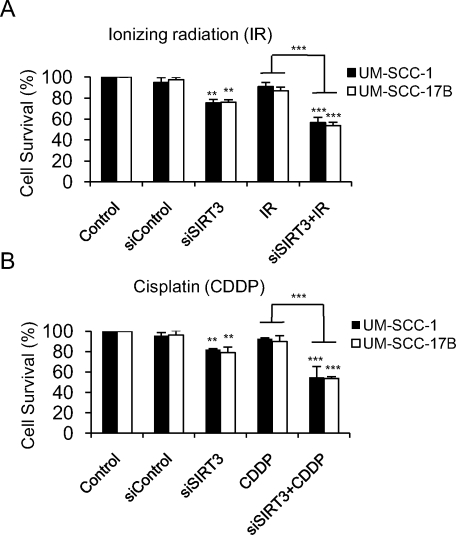
Sirtuin-3 (SIRT3) down-regulation enhances the sensitivity of oral squamous cell carcinoma (OSCC) to radiation and cisplatin-induced cytotoxicity. OSCC cells (UM-SCC-1 and UM-SCC-17B) were either untransfected or transfected with small interfering SIRT3 (siSIRT3) (150 nM) and were treated with either (A) ionizing radiation (2.5 grays) or (B) cisplatin (20 μM) for 24 hours, and cytotoxicity was determined by using the QUANT Cell Proliferation Assay Kit (Invitrogen). **P ≤ .01; ***P ≤ .001.

Because radiation is the primary treatment modality in head and neck cancer, and cisplatin is 1 of the first chemotherapeutic drugs used to treat OSCC,^26^ we investigated whether SIRT3 down-regulation with siRNA enhances the sensitivity of OSCC cells to radiation and cisplatin. Cytotoxicity assays were performed first to optimize ionizing radiation (IR) and cisplatin (CDDP) doses and determine the 50% inhibitory concentration for both treatments in all cell lines (data not shown). Indeed, down-regulation of SIRT3 with siRNA enhanced the sensitivity of the UM-SCC-1 and UM-SCC-17B cell lines to both treatments compared with untreated controls and with cells that were treated radiation or cisplatin alone (Fig. 4).

### SIRT3 Down-Regulation Reduces Oral Squamous Cell Carcinoma Tumor Burden in Vivo

**Figure 5:**
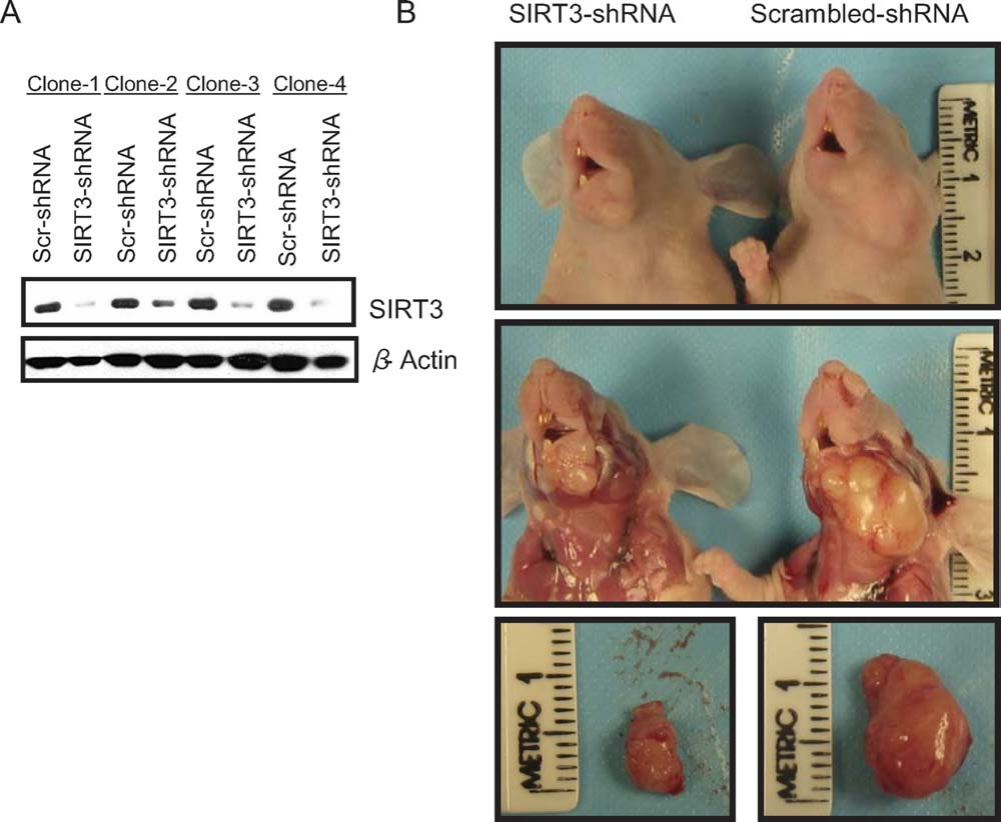
Sirtuin-3 (SIRT3) down-regulation reduces oral squamous cell carcinoma (OSCC) tumor burden in vivo. (A) These immunoblots show SIRT3 expression levels in 4 clones from UM-SCC-17B cell lines that were stably transfected with scrambled-short hairpin RNA (Scr-shRNA) or SIRT3-shRNA after 10 days of selection using Puromycin. β-Actin served as loading control. (B) Mice were injected with OSCC cells that stably expressed Scr-shRNA or SIRT3-shRNA. Images show (*Top*) the superficial growth of tumors in the head and neck region of the mice, (*Middle*) dissected tumors in situ, and (*Bottom*) dissected and isolated tumors from the 2 groups.

To further demonstrate the role of SIRT3 in OSCC carcinogenesis in vivo, we used a murine floor-of-mouth model that mimics human OSCC.^23^, ^27^ The UM-SCC-17B cell line was selected for examination in this model because of its highly aggressive nature and its resistance to radiation therapy, and because it commonly produces tumors in this in vivo model.^23^ The UM-SCC-17B cell line was used to produce stable clones that expressed down-regulated levels of SIRT3 using SIRT3-shRNA, and scrambled-shRNA was used for controls (Fig. 5A). SIRT3-shRNA (Clone 4) and scrambled-shRNA (Clone 1) were selected for the in vivo injections. In agreement with our in vitro data, our mouse model data indicated that the down-regulation of SIRT3 in OSCC cells significantly inhibited tumor growth in vivo (Fig. 5B). Specifically, the mean tumor volumes for the SIRT3 down-regulated group and the control group were 26.948 mm^3^ and 112.325 mm^3^, respectively (*P* ≤ .001) (Fig. 5B, Table 2).

**Table 2:** Summary of Tumor Volume in Mice Injected With UM-SCC-17B Cells that Stably Expressed Scrambled Short Hairpin RNA (shRNA) or Sirtun-3 shRNA^a^

Animal	Tumor Volume, mm^3^	
	Scrambled shRNA	SIRT3-shRNA
1	190.8	21.1
2	186.9	35.75
3	100.7	32.8
4	165.1	20.1
5	124.62	26
6	92.23	28.5
7	71.3	37.4
8	64.4	40.63
9	64	15.3
10	63.2	11.9
Mean volume	112.325	26.948^b^

SIRT3 indicates sirtuin-3.

a Statistical analysis: independent *t* test with unequal variances.

b Significant difference (*P* ≤ .001).

## DISCUSSION

Here, we report for the first time a role for sirtuins and, specifically, for SIRT3 in OSCC carcinogenesis. There is emerging evidence of a role for several sirtuins in carcinogenesis.^4^ However, to our knowledge, their role in oral cancer has not been investigated previously. Thus, we explored their potential role in OSCC by evaluating their expression levels in several OSCC cell lines compared with normal human oral keratinocytes. Our initial finding that SIRT3 was overexpressed in OSCC cells compared with normal human oral keratinocytes led us to hypothesize that SIRT3 may play a role in OSCC carcinogenesis. Thus, to further explore the clinical relevance of this overexpression, we used tissue microarray analyses of OSCC tissues. The high expression levels of SIRT3 in OSCC tissues compared with normal tissues further supported a role for SIRT3 in oral cancer carcinogenesis. Sirtuin inhibitors, such as sirtinol and NAM, which have been used to inhibit cell growth in several types of cancers, such as breast and lung cancers,^24^ also inhibited cell growth and proliferation and induced apoptosis in several OSCC cell lines.

Although some investigators have suggested a proapoptotic role for SIRT3,^28^-^30^ others have suggested a prosurvival role.^20^-^22^, ^31^ In addition, some reports suggest that SIRT3 is exclusively a mitochondrial protein^12^-^18^; however, others have reported that SIRT3 can be observed in the cytosol and nucleus during different cellular events.^20^, ^32^, ^33^ Those reports underscore the complexity of the biologic functions of sirtuins, which may differ according to their tissue of origin or cancer type. For example, SIRT1 functions in the neural system to promote neural cell survival and to protect against genomic toxicity^30^; however, this may not be true in all cancer types. There is a discrepancy in the literature regarding the role of SIRT1 in cancer.^7^, ^8^, ^24^ After investigating the effect of SIRT1 in several cancer types, Stunkel et al^34^ concluded that the function of SIRT1 in cancer is cell-context-dependent and that the role of SIRT1 can be independent of its deacetylase function. This cell specificity also may be true for SIRT3. When SIRT3 is overexpressed in cardiomyocytes, it increases stress resistance and plays a protective role against cell death and apoptosis.^20^ SIRT3 also seems to play a protective role against cardiac hypertrophy and heart failure.^35^ Furthermore, SIRT3 is overexpressed in breast cancer, and it was shown to modulate p53 activity, preventing growth arrest and senescence in bladder carcinoma cells.^19^, ^22^ Because our current findings indicate that SIRT3 is overexpressed in OSCC tissues and cells (Fig. 1), we surmised that SIRT3 also plays a prosurvival role in oral cancer.

Our current data indicate that SIRT3 levels are elevated in head and neck cancer and that the suppression of SIRT3 levels reduces several tumorigenic parameters in vitro and in vivo. In contrast, in a recent study, other investigators speculated about the role of SIRT3 in head and neck cancer by reviewing published gene array data from another source and observed that SIRT3 levels are decreased in head and neck cancer.^36^ Those findings are contradictory to findings in the current report; however, it must be noted that those studies were limited, because they reviewed only gene array studies and did not include the kinds of full functional assessment of SIRT3 in vitro and in vivo that were part of the current study.

The down-regulation of SIRT3 inhibited colony formation and induced apoptosis in OSCC cells. In addition, we investigated whether SIRT3 could modulate the sensitivity of OSCC cells to both IR and cisplatin treatment. We tested UM-SCC-1 cells and UM-SCC-17B cells, both of which are highly resistant to radiation,^37^, ^38^ and the former cells also are resistant to cisplatin treatment (data not shown). Our data demonstrated that SIRT3 down-regulation sensitized OSCC cells to both IR and cisplatin treatment, indicating that SIRT3 is important in the modulation of OSCC-induced resistance to both treatments. Thus, targeting SIRT3 to induce OSCC cell cytotoxicity in patients who have high SIRT3-expressing tumors may be advantageous, because lower doses of treatment would be required. Furthermore, SIRT3 may serve as an adjunctive target to improve the efficacy and decrease the side effects of conventional treatments. In addition, although SIRT3-deficient mice had hyperacetylated mitochondrial proteins, they were surprisingly healthy, with normal bone mineral density and an unremarkable phenotype compared with wild-type SIRT3 mice.^18^ This suggests that targeting SIRT3 in oral cancer may be less toxic to normal cells versus cancer cells. Parenthetically, gene deletions of other sirtuins produce more aggressive and lethal phenotypes.^39^, ^40^

To further demonstrate the important role of SIRT3 in OSCC carcinogenesis, we used an in vivo murine floor-of-mouth model that mimicked human OSCC.^23^, ^27^ This model has the advantage that tumors injected into the floor of the mouth behave like human head and neck squamous cell carcinoma, growing in the same environment, growing aggressively, and invading surrounding tissues. It is noteworthy that our data indicate that tumor cells with low levels of SIRT3 grow slower, produce smaller tumors, and have less tumor volume compared with controls (Fig. 3, Table 1). These findings support the in vitro data and indicate that SIRT3 plays an important role in oral cancer carcinogenesis in vivo.

In summary, our findings reveal a novel role for SIRT3 in oral cancer carcinogenesis as a modulator of cell proliferation and survival, supported by in vitro and in vivo data. This implicates SIRT3 as a new potential therapeutic target for treating oral cancer.
